# Pre-Wetting Reduces Blood Component Deposition on Polyvinyl Alcohol-Coated Poly-ε-Caprolactone Nanofiber Grafts

**DOI:** 10.3390/bioengineering13070737

**Published:** 2026-06-25

**Authors:** Masahiro Tsutsui, Takumi Yoshida, Daisuke Naruse, Shingo Kunioka, Daisuke Koga, Yuta Kikuchi, Naohiro Wakabayashi, Hiroyuki Kamiya, Kyohei Oyama

**Affiliations:** 1 Department of Cardiac Surgery, Asahikawa Medical University, Asahikawa 078-8510, Japan; mtsutsui@asahikawa-med.ac.jp (M.T.); s_kunioka@asahikawa-med.ac.jp (S.K.); ykikuchi0613@gmail.com (Y.K.); wkbys_1234@hotmail.co.jp (N.W.); kamiya@asahikawa-med.ac.jp (H.K.); 2 Life Materials Development Section, Human Life Technology Research Institute, Toyama Industrial Technology Research and Development Center, Toyama 939-1503, Japan; yoshida@itc.pref.toyama.jp; 3 Business Development Section, Business Development and Quality Control Department, Iaazaj Holdings Co., Ltd., Toyama 932-0398, Japan; d-naruse@ichiamiaz.co.jp; 4 Department of Microscopic Anatomy and Cell Biology, Asahikawa Medical University, Asahikawa 078-8510, Japan; daisukek@asahikawamed.ac.jp

**Keywords:** small-diameter vascular graft, nanofiber graft, poly-ε-caprolactone, polyvinyl alcohol, surface hydration, hemocompatibility

## Abstract

Hydrophilic surface modification is widely investigated as a strategy to improve the hemocompatibility of small-diameter vascular grafts. We previously developed a polyvinyl alcohol-coated poly-ε-caprolactone nanofiber graft (PVA–PCL graft) and showed that the PVA coating improved graft hydrophilicity and mechanical properties. However, whether this coating provides an in vivo advantage over uncoated PCL grafts remains unclear. In addition, the influence of pre-implantation surface hydration on the function of hydrophilic grafts has not been fully examined. In this study, we first compared PVA–PCL and uncoated PCL grafts in a rat abdominal aorta implantation model and found no statistically significant difference in patency rate between the graft types. We then examined whether pre-wetting enhanced the anti-fouling function of the PVA coating. In vitro whole-blood flushing assays demonstrated that pre-wetting markedly reduced blood component deposition on PVA–PCL grafts, but this effect did not translate into detectable improvements in patency or tissue regeneration in the rat model. These findings indicate that pre-wetting effectively enhances the in vitro anti-fouling behavior of PVA–PCL grafts and may serve as a simple strategy to optimize the functional surface state of hydrophilic coatings. Further studies are needed to determine whether this in vitro improvement can be translated into meaningful enhancement in graft performance in vivo.

## 1. Introduction

Small-diameter surgical revascularization procedures, such as coronary artery bypass grafting (CABG) and distal bypass for lower-extremity peripheral arterial disease, rely primarily on autologous vessels. However, suitable autologous grafts are not always available because of poor vessel quality or insufficient length [1]. In this setting, clinically reliable synthetic small-diameter vascular grafts are still lacking, mainly because small-caliber grafts are prone to thrombosis, insufficient endothelialization, and intimal hyperplasia, particularly under low-flow conditions [2,3]. Consequently, various vascular tissue engineering approaches have been explored to develop clinically applicable off-the-shelf small-diameter vascular grafts [4,5]. Nevertheless, the development of such grafts remains an unmet clinical need.

Synthetic polymers are widely investigated in vascular tissue engineering because of their advantages in cost, scalability, and stable supply [6]. Among them, poly-ε-caprolactone (PCL) is a biocompatible and biodegradable polymer, and electrospun PCL nanofibers have attracted attention as scaffold materials for small-diameter vascular graft fabrication. However, the hydrophobic nature of PCL is a major concern, as hydrophobic blood-contacting surfaces can promote adsorption of blood components and platelet activation, potentially leading to thrombosis [7,8]. To address this disadvantage, we previously developed a PCL nanofiber graft coated with the hydrophilic polymer polyvinyl alcohol (PVA) (PVA–PCL graft) with the aim of improving hemocompatibility. PVA was selected because its abundant hydroxyl groups confer high hydrophilicity and can promote the formation of a hydrated interfacial layer. Such hydrophilic interfaces have been reported to reduce non-specific protein adsorption and platelet adhesion, which are key early events in thrombus formation on blood-contacting materials [9,10]. In our previous study, a PVA coating improved the mechanical properties and handling of the PCL graft, increased surface hydrophilicity, and suppressed platelet adsorption in experiments using platelet-rich plasma [11]. Moreover, the PVA–PCL graft demonstrated patency for 8 weeks with autologous tissue-like regeneration in a rat implantation model [11]. However, despite these improvements in material properties, it remains unclear whether the PVA coating provides a measurable in vivo advantage over an uncoated PCL graft, particularly in terms of graft patency and tissue regeneration in an animal model.

In addition to the material composition itself, graft performance may depend on the pre-implantation condition [12,13]. Hydrophilic materials are known to reduce non-specific adsorption of biological components, including proteins and platelets, and this anti-fouling behavior has been attributed to the formation and retention of a hydration layer at the material–blood interface [14,15]. From this perspective, a PVA-coated surface may function optimally when it is sufficiently hydrated at the time of blood contact. If a simple and clinically feasible preparation step, such as pre-wetting, could maximize the hemocompatibility conferred by a hydrophilic coating, this strategy could improve translational feasibility without adding complexity to graft fabrication.

Therefore, this study aimed to address two previously unresolved questions: (1) whether a PVA coating provides a measurable in vivo advantage over uncoated PCL grafts in terms of patency and tissue regeneration and (2) whether a simple pre-wetting preparation influences the functional performance of the PVA–PCL graft.

## 2. Materials and Methods

### 2.1. Nanofiber Graft Preparation

The PVA-coated PCL nanofiber graft (PVA–PCL graft) was prepared as previously described [11]. Briefly, electrospinning was performed using a custom-built electrospinning apparatus equipped with a high-voltage power supply (Green Techno, Kawasaki, Japan, GT80P). A PCL nanofiber sheet was electrospun from a 10% (*w*/*v*) poly(ε-caprolactone) solution (PCL; Mw 80,000; Sigma-Aldrich, St. Louis, MO, USA, Cat. No. 440744) dissolved in a mixture of N,N-dimethylformamide (DMF; Sigma-Aldrich, Cat. No. 07-4440-5, pure grade ≥ 99.0%) and tetrahydrofuran (THF; Kishida Chemical, Oosaka, Japan, Cat. No. 010-76815, pure grade 99.0%) (3:7, *w*/*w*) under the following conditions: an applied voltage of 20 kV, a tip-to-collector distance of 20 cm, a flow rate of 1.7 mL/h, and a collector rotation speed of 20 rpm. The resulting nanofiber sheet was cut into 20 mm wide strips and wrapped around a 1 mm diameter polytetrafluoroethylene (PTFE) mandrel to form three layers. After removing the PTFE mandrel, the resulting tubular scaffold was defined as the uncoated PCL nanofiber graft (PCL graft). To generate the PVA-coated graft, a coating solution was prepared by mixing a commercially available 5% (*w*/*v*) PVA (Kaneyo Soap, Tokyo, Japan, 606021-A) aqueous solution with ethanol (Kishida Chemical, Cat. No. 000-28556, pure grade 99.5%) and water at a weight ratio of 1:1:1. One milliliter of the coating solution was drawn into a 5 mL syringe connected to a tapered nozzle; this nozzle was inserted into the PCL graft and the coating solution was slowly extruded through the graft lumen. This coating procedure was performed twice, using 1 mL of coating solution each time. Air was then passed through the graft using an empty syringe to remove the excess solution remaining in the graft. Finally, the grafts were dried overnight at room temperature (approximately 25 °C) to obtain the PVA–PCL graft. Approximately 58.6 mg of the PVA coating was used per gram of the dry PVA–PCL graft, corresponding to approximately 5.9 wt% of the final PVA–PCL graft weight.

### 2.2. Nanofiber Graft Implantation and Harvest In Vivo

All animal experiments were approved by the Institutional Animal Care and Use Committee of Asahikawa Medical University (Approval Nos. R4-030, R5-028, R6-094, and R7-042; Dates of Approval: 31 January 2023, 31 January 2024, 19 January 2025, and 10 January 2026, respectively). Male Wistar rats (8–10 weeks old; Charles River, Japan) were anesthetized with 5% isoflurane for induction and maintained with 2–3% isoflurane delivered via a small-animal anesthesia machine. A midline laparotomy was performed to expose the abdominal aorta, and the inferior vena cava was carefully dissected free from the aorta. The infrarenal aorta was clamped just below the left renal artery and above the aortic bifurcation. The aorta was transected and replaced with a nanofiber graft (PCL graft or PVA–PCL graft) (inner diameter, 1 mm; length, ~10 mm), and this graft was anastomosed to the aorta using an end-to-end technique using running 10-0 polypropylene sutures. After removing the clamps, hemostasis was confirmed and the abdominal incision was closed. Rats were allowed free access to food and water, and no antiplatelet or anticoagulant medications were administered during the follow-up period.

At the study’s endpoint, graft patency was assessed under general anesthesia by confirming pulsatile blood flow distal to the graft. The grafts were then flushed with PBS and perfusion-fixed with 4% paraformaldehyde (PFA) via intracardiac infusion through the left ventricular apex, followed by graft explantation.

### 2.3. In Vitro Whole-Blood Flushing Assay

With approval from the Institutional Review Board of Asahikawa Medical University (Approval Nos. 21,166 and 24153; Dates of Approval: 18 March 2022 and 2 April 2025, respectively), peripheral blood (20 mL) was collected from three healthy volunteers, after written informed consent, into standard K2EDTA-coated blood collection tubes to prevent coagulation. Samples were kept at room temperature and used within 1 h of collection. PVA–PCL grafts (inner diameter, 3 mm; length, 20 mm) were prepared and assigned to either a pre-wet (wet) or non-pre-wet (dry) condition. For the wet condition, grafts were immersed in Milli-Q water for 10 min immediately before testing; dry grafts were used without pre-wetting. Human whole blood (2.5 mL) was flushed through each graft three times using a 2.5 mL syringe at approximately 1 mL/s, and the grafts were then flushed three times with phosphate-buffered saline (PBS) using a 20 mL syringe at approximately 1 mL/s. After flushing, grafts were opened longitudinally to expose the luminal surface for macroscopic inspection and then fixed in 2% glutaraldehyde for subsequent scanning electron microscopy.

### 2.4. Histological Analysis

Explanted grafts were further fixed overnight in 4% paraformaldehyde (PFA). After washing with PBS, grafts were cut longitudinally into two halves, each containing both the proximal and distal anastomotic native vessels. Samples were embedded in optimal cutting temperature (OCT) compound (Sakura Finetek, Tokyo, Japan) and stored at −80 °C. Frozen specimens were sectioned at 5 µm thickness using a cryostat at −20 °C and mounted on glass slides.

Hematoxylin and eosin (H&E) staining was performed using a standard protocol to evaluate tissue morphology and cellular repopulation of the graft lumen. Briefly, sections were washed in PBS for 5 min to remove OCT, stained with hematoxylin solution (FUJIFILM, Tokyo, Japan 131-09665) for 4 min, and rinsed under running water for 6 min. Sections were then stained with 1% eosin solution (FUJIFILM, Tokyo, Japan 051-06515) for 2 min and rinsed three times with 70% ethanol. Slides were mounted with Marinol 750 (Muto Pure Chemicals, Tokyo, Japan).

For immunofluorescence staining, heat-induced antigen retrieval was performed in Tris–EDTA buffer (10 mM Tris base, 1 mM EDTA, 0.05% Tween 20, pH 9.0) at 98 °C for 1 h. Sections were blocked with 1% bovine serum albumin (BSA) in PBS and incubated overnight at 4 °C with primary antibodies against CD31 (R&D Systems, Minneapolis, MN, USA, AF3628; 1:200) and α-smooth muscle actin (Cell Signaling Technology, Danvers, MA, USA, #56856; 1:250). Alexa Fluor 488-conjugated anti-goat IgG (Thermo Fisher Scientific, A-11055) and Alexa Fluor 555-conjugated anti-mouse IgG (Thermo Fisher Scientific, Tokyo, Japann, A-31570) were applied as secondary antibodies for 1 h at room temperature. Nuclei were counterstained with Hoechst 33,342 (FUJIFILM, Tokyo, Japan, 346-07951).

Images of H&E- and immunofluorescence-stained sections were acquired using an all-in-one fluorescence microscope (Keyence, Oosaka, Japan, BZ-X810).

### 2.5. Scanning Electron Microscopy Analysis

For conductive staining, graft specimens fixed in 2% glutaraldehyde were immersed in 1% tannic acid in 0.1 M phosphate buffer (PB) for 1 h, rinsed in PB for 1 h, and post-fixed in 1% osmium tetroxide in 0.1 M PB for 1 h. Specimens were then dehydrated through a graded ethanol series (70%, 80%, 90%, and 95%; 30 min each). After dehydration, specimens were dried in a critical point dryer (Hitachi, Tokyo, Japan, HCP-2). The dried specimens were mounted on aluminum bases, coated with platinum–palladium using an ion-sputter coater (Hitachi, Tokyo, Japan, E1010), and observed in a scanning electron microscope (Hitachi, Tokyo, Japan, S-4100).

### 2.6. Statistical Analysis

All statistical analyses were performed with EZR version 1.54 [16]. All nominal variables were analyzed with Fisher’s exact test, and *p* < 0.05 was considered statistically significant.

## 3. Results

### 3.1. Comparison of PVA-Coated and -Non-Coated PCL Graft

**Figure 1:**
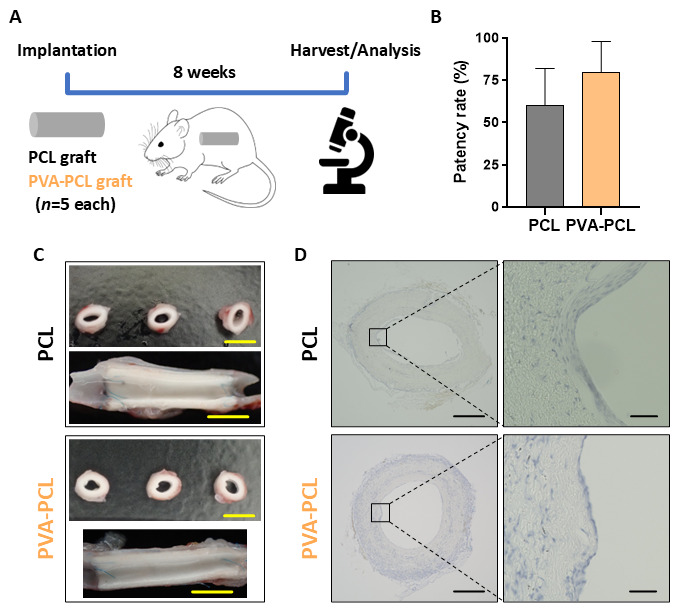
**In vivo comparison of PVA–PCL and PCL grafts.** The yellow text and graph represent the PVA-PCL graft, while the black text and graph represent the PCL graft. (**A**) **Schematic of the experimental design.** PVA–PCL and PCL grafts of the same size were prepared and implanted into the abdominal aorta of rats (n = 5 per group). Grafts were harvested at 8 weeks for assessment of patency and autologous tissue regeneration. (**B**) **Patency rate at 8 weeks.** Patency was evaluated by confirming pulsatile blood flow distal to the graft and by macroscopic inspection. (**C**) **Macroscopic images of explanted grafts.** Representative cross-sectional (**upper**) and longitudinal (**lower**) views are shown. Scale bars, 2 mm. (**D**) **Hematoxylin and eosin (H&E) staining of explanted grafts.** Representative low-magnification (**left**) and high-magnification (**right**) images are shown. Scale bars, 500 µm (**left**) and 50 µm (**right**).

We previously reported that the PVA coating increases the hydrophilicity and improves the mechanical properties of electrospun PCL nanofiber vascular grafts (PCL grafts). However, the in vivo efficacy of PVA-coated PCL grafts (PVA–PCL grafts) compared with uncoated PCL grafts has not been evaluated. Therefore, we examined whether this PVA coating provides advantages in patency in vivo by directly comparing PVA–PCL and PCL grafts (Figure 1).

These two grafts (inner diameter, 1 mm; length, 10 mm) were implanted into the abdominal aorta of rats (n = 5 per group) and harvested at 8 weeks post implantation for analysis (Figure 1A). The patency rate was 60% (3/5) in the PCL group and 80% (4/5) in the PVA–PCL group, with no statistically significant difference between groups (*p* = 1.0) (Figure 1B). Macroscopic and histological analyses revealed no clear differences between groups; both graft types exhibited a patent lumen and tissue regeneration along the graft wall (Figure 1C,D). Collectively, the PVA coating did not confer a detectable advantage over uncoated PCL grafts in terms of patency in this model.

### 3.2. Pre-Wetting Reduces Blood Component Deposition on PVA–PCL Grafts

In our previous study, the PVA coating increased the hydrophilicity of PCL grafts. Hydrophilic surfaces are generally associated with reduced non-specific adsorption of biocomponents, and this behavior has been attributed to the formation of a hydration layer at the blood–material interface [17]. However, in the present study, PVA–PCL grafts did not show a statistically significant improvement in patency compared with uncoated PCL grafts (Figure 1). Because the hemocompatible function of a hydrophilic coating may depend on its hydration state at the time of blood contact, we hypothesized that pre-wetting of PVA–PCL grafts would enhance their anti-fouling performance. To test this possibility, we first examined the effect of pre-wetting on blood component deposition on PVA–PCL grafts in vitro.

**Figure 2:**
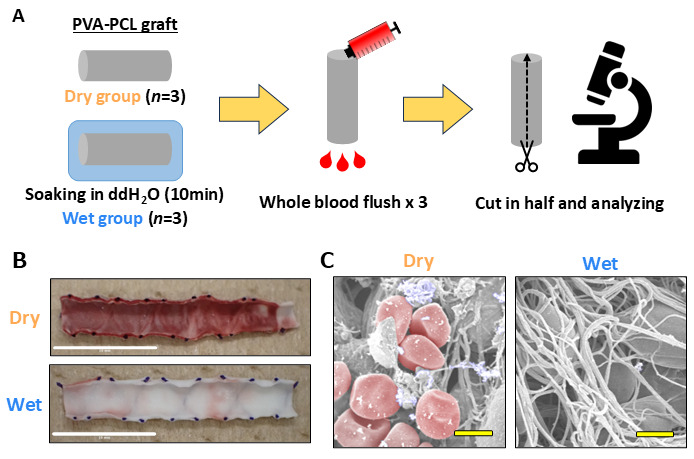
Effect of pre-wetting on blood component deposition on PVA–PCL grafts in vitro. Yellow indicates the Dry Group, and blue indicates the Wet Group. (**A**) **Schematic of the experimental design**. PVA–PCL grafts were either immersed in double-distilled water for 10 min (wet group) or used without pre-wetting (dry group). Human whole blood (2.5 mL) was flushed through each graft three times using a syringe. Grafts were then opened longitudinally to evaluate luminal deposition, and the luminal surface was examined. (**B**) **Macroscopic images of graft lumens after blood flushing**. Representative images of the luminal surfaces of wet and dry grafts are shown. Scale bars, 10 mm. (**C**) **Scanning electron microscopy of graft lumens after blood flushing**. Representative SEM images of the luminal surfaces of wet and dry grafts are shown. For clarity, adherent erythrocytes and granular blood-derived deposits in the dry group are pseudo-colored red and blue, respectively. Scale bars, 5 μm.

PVA–PCL grafts (inner diameter, 3 mm; length, 20 mm) were prepared and either immersed in Milli-Q water for 10 min (wet group) or used without pre-wetting (dry group) (Figure 2A). Human whole blood (2.5 mL) was flushed through each graft three times using a syringe, after which the grafts were opened longitudinally for evaluation of luminal deposition (Figure 2A). Macroscopic observation revealed clear differences between groups: the luminal surface of dry grafts appeared red-stained, suggesting deposition of erythrocyte-rich components, whereas wet grafts showed minimal visible staining (Figure 2B). Scanning electron microscopy further supported these observations. Dry grafts exhibited extensive deposition of granular material and adherent blood cells, whereas wet grafts showed markedly reduced surface deposits (Figure 2C). Taken together, these results indicate that pre-wetting reduces blood component deposition on PVA–PCL grafts in vitro.

### 3.3. Pre-Wetting Has No Effect on In Vivo Graft Performance

**Figure 3:**
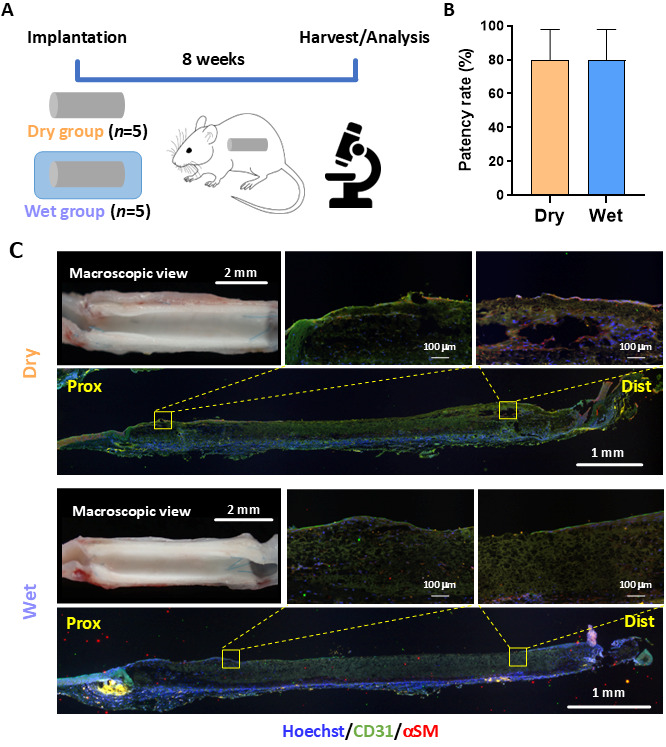
**Effect of pre-wetting on PVA–PCL graft performance in vivo.** (**A**) **Schematic of the experimental design.** PVA–PCL grafts were either immersed in phosphate-buffered saline (PBS) for 5 min before implantation (wet group) or implanted without pre-wetting (dry group). Grafts were implanted into the abdominal aorta of rats (n = 5 per group) and harvested at 8 weeks for assessment of patency and autologous tissue regeneration. (**B**) **Patency rate at 8 weeks.** Patency was evaluated by confirming pulsatile blood flow distal to the graft and by macroscopic inspection. (**C**) **Macroscopic and immunofluorescence analyses of explanted grafts.** Representative macroscopic images are shown (upper left in each group). Scale bars, 2 mm. Representative immunofluorescence images at low magnification (upper middle and right) and high magnification (lower) are shown. CD31 (green), α-smooth muscle actin (red), and nuclei (blue). Scale bars, 100 µm (low magnification) and 1 mm (high magnification).The yellow squares indicate which parts of the low-magnification image have been enlarged to high magnification.

Because pre-wetting reduced blood component deposition on PVA–PCL grafts in vitro (Figure 2), we next evaluated whether pre-wetting improves graft performance in vivo using a rat abdominal aorta implantation model (Figure 3). PVA–PCL grafts (inner diameter, 1 mm; length, 10 mm) were either immersed in phosphate-buffered saline (PBS) for 5 min before implantation (wet group) or implanted without pre-wetting (dry group). Grafts were implanted into the abdominal aorta of rats and harvested at 8 weeks post implantation for assessment of patency and tissue regeneration (Figure 3A). The patency rate was 80% (4/5) in both groups, with no statistically significant difference (*p* = 1.0) (Figure 3B). Macroscopic observation of explanted grafts revealed no clear differences between groups; the luminal surface appeared white and smooth in both wet and dry conditions (Figure 3C). Immunofluorescence analyses further demonstrated comparable autologous vessel-like regeneration in both groups, including endothelial coverage and smooth-muscle-cell-positive neointimal formation along the graft wall (Figure 3C).

Thus, although pre-wetting reduced blood component deposition in vitro, this preparation step did not result in detectable improvements in patency or tissue regeneration in this rat implantation model.

## 4. Discussion

In the present study, we performed two sequential evaluations to clarify the functional significance of PVA-coating and pre-wetting electrospun PCL nanofiber grafts. Although no functional advantage was detected in the rat implantation model used in this study, a key finding was that pre-wetting clearly reduced blood component deposition on PVA–PCL grafts in vitro. This finding is consistent with the possibility that prior surface hydration may facilitate the anti-fouling function of the PVA coating.

### 4.1. Pre-Wetting as Optimization of the Functional Surface State of PVA Coating

Hydrophilic surfaces are generally considered to suppress the non-specific adsorption of blood components, including proteins, platelets, and blood cells, and therefore have been widely investigated for application to blood-contacting medical devices [14]. One proposed mechanism underlying this effect is the formation of intermediate water at the material–blood interface, which may reduce direct contact between biological components and the polymer surface [14,17]. However, previous studies on hydrophilic or low-fouling biomaterials have mainly discussed anti-fouling behavior from the viewpoints of surface chemistry and interfacial hydration/water structures, whereas the practical influence of the hydration state immediately before blood exposure has been less thoroughly addressed [9,18]. To our knowledge, whether pre-wetting of a hydrophilically modified small-diameter vascular graft can suppress blood component deposition has not been directly examined. This represents a practical gap because hydrophilic coatings may not fully express their anti-fouling properties when they are exposed to blood in a dry or insufficiently hydrated state. Based on these concepts, we previously developed a PVA-coated PCL nanofiber graft and demonstrated that the PVA coating increased the hydrophilicity of the PCL graft [11]. In the present study, we further considered that, although PVA is itself a hydrophilic material, its anti-fouling potential might be more effectively expressed when the coated surface is already hydrated before exposure to blood. Indeed, pre-wetting clearly reduced blood component deposition on PVA–PCL grafts in vitro (Figure 2). Although the present study did not directly characterize the interfacial water structure of the PVA-coated surface, our findings are consistent with the idea proposed in previous studies that intermediate water suppresses the adsorption of biological components [14,17]. Thus, the anti-fouling effect observed in pre-wet PVA–PCL grafts may reflect more effective expression of the intrinsic hemocompatible properties of the PVA coating through prior surface hydration. This concept may not be limited to PVA–PCL grafts alone. Rather, simple pre-wetting may be a practical approach to help express the anti-fouling potential of hydrophilic materials or surface coatings, a possibility that should be examined in future studies across a broader range of biomaterial systems.

### 4.2. Discrepancy in PVA–PCL Graft Performance Between In Vitro and In Vivo Evaluations

Despite the clear in vitro effect of pre-wetting, no improvement in patency or autologous vessel-like regeneration was observed in vivo. One possible explanation is that the difference between pre-wet and non-pre-wet PVA–PCL grafts may have been reduced during the surgical procedure itself. Even grafts implanted without intentional pre-wetting may have become hydrated through exposure to the operative field, blood, or surrounding moisture, such that the effective difference in hydration state between the two groups at the time of blood contact was smaller in vivo than in the controlled in vitro setting. In addition, we speculate that the rat abdominal aorta implantation model itself may not be sufficiently sensitive to detect modest differences in antithrombogenic performance. This model is useful for evaluating graft biocompatibility, tissue regeneration, and surgical handling in vivo. However, it also has physical and hemodynamic limitations for evaluating patency differences, including the use of relatively short graft segments in a high-flow, high-pressure arterial environment. Under these conditions, grafts may remain patent as long as the anastomosis is technically adequate. Consistent with this interpretation, previous studies using this model have frequently reported favorable patency rates for small-diameter vascular grafts [15,19,20]. Although this observation does not directly prove a limitation of the model, it may suggest that this model is less suitable for discriminating subtle functional differences in graft hemocompatibility.

Therefore, we interpret the absence of a detectable in vivo benefit in the present study as not necessarily indicating that PVA-coating or pre-wetting is ineffective. Rather, the present findings suggest that a gap remains between simplified in vitro evaluations and in vivo graft performance. Establishing evaluation systems in which in vitro and in vivo findings more closely correspond to each other will be important for more accurately assessing the functional value of surface modification and pre-conditioning strategies.

## 5. Limitations

This study has several limitations. First, the mechanism underlying the discrepancy between the in vitro anti-fouling effect of pre-wetting and the absence of a detectable in vivo difference was not determined. Although hydration of the graft during the surgical procedure and the limited sensitivity of the rat abdominal aorta model may have contributed to this discrepancy, these possibilities were not directly tested in the present study.

Second, quantitative analysis of the immunofluorescence data was not performed. Although CD31- and αSMA-positive signals were detectable in the neointimal tissue formed along the graft wall, reliable quantitative analysis was technically difficult because of background fluorescence from the PVA–PCL scaffold and partial loss or detachment of the fragile neointimal tissue during section preparation. Therefore, the immunofluorescence findings were evaluated qualitatively.

## 6. Conclusions

In conclusion, pre-wetting reduced blood component deposition on PVA–PCL nanofiber grafts in vitro; however, this effect did not translate into improved in vivo outcomes. Further studies using refined in vitro assays and alternative in vivo evaluation strategies and animal models will be required to clarify why the in vitro anti-fouling effect was not reflected in vivo. Elucidating the determinants of this in vitro–in vivo discrepancy will enable rational optimization of surface and pre-conditioning strategies to more reliably predict and improve the long-term patency of clinically translatable small-diameter vascular grafts.

## Data Availability

The data presented in this study are available on request from the corresponding author.
